# Causal splicing variants revealed by deep-learning integration of single-cell sQTL mapping under influenza infection

**DOI:** 10.21203/rs.3.rs-8408992/v1

**Published:** 2026-01-06

**Authors:** Liuyang Wang, Guinevere Connelly, Trisha Dalapati, Angela Jones, Benjamin Schott, Joseph Trimarco, Nicholas Heaton, Dennis Ko

**Affiliations:** Duke University; Duke University; Duke University; Duke University; Duke University; Duke University; Duke University; Duke University

**Keywords:** sQTL, scHi-HOST, GWAS, influenza A virus, AI, PARP2, rs2297616, OAS1, U2AF1L4

## Abstract

**Background:**

Fulfilling the promise of human genetics in elucidating disease requires identifying causal variants and genes underlying genetic association signals. Molecular quantitative trait locus (molQTL) analyses, e.g. expression QTL (eQTL) and splicing QTL (sQTL), link genetic variants to intermediate molecular phenotypes, but pinpointing causal variants and their regulatory effects remains challenging. Here, we integrate sQTL analysis with deep-learning-based splicing effect annotation to identify causal genetic variants and elucidate their functional mechanisms affecting human phenotypes.

**Results:**

Using a single-cell GWAS method (scHi-HOST) on 96 lymphoblastoid cell lines (LCLs) with and without influenza A virus (IAV) infection, we discovered ~ 43,000 sQTLs associated with 217 genes after IAV infection. Integrating sQTLs with AI splice prediction, we uncovered 76 likely causal variants that affect cis-acting molecular splicing components (5’ donor, 3’ acceptor), supported by further computational analysis. Among these, we experimentally validated a causal sQTL signal affecting poly (ADP-ribose) polymerase 2 (PARP2). The causal variant, rs2297616, alters the 5’ splice donor site in the second intron of *PARP2*, resulting in two protein isoforms differing by 13 amino acids. The derived A allele was associated with the longer protein isoform and increased IAV levels in LCLs. CRISPR editing validated the causal effect of this variant on both protein length and IAV infection. Lastly, these 76 putative causal sQTLs were further linked to over a hundred GWAS traits, including many variants associated with autoimmune diseases.

**Conclusions:**

Our work provides a catalog of causal sQTL with direct splicing impacts, providing causal mechanistic insights from genotype to disease susceptibility.

## Background

Deciphering the causal genetic basis of disease susceptibility is central to human genetics and precision medicine[1]. Molecular quantitative trait loci (molQTLs), e.g. expression QTLs (eQTLs) and splicing QTLs (sQTLs), provide potential mechanistic insight by linking genetic variants to intermediate molecular phenotypes[2–4]. Further, joint analysis of GWAS and molQTL has shown promise in narrowing down candidate causal SNPs and their underlying causal genes[5, 6].

Two broad categories of joint analyses have been widely used to prioritize disease-associated genes. The first, exemplified by PrediXcan[7] and transcriptome-wide association studies[8], estimate association of imputed gene expression and disease outcomes. A complementary approach relies on colocalization to infer shared causal genetic signals between GWAS and molQTL in a specific genomic region (e.g. coloc[9], RTC[10], Sherlock[11], and eCAVIAR[12]). Despite their success in pinpointing causal factors, the first type of method focuses on identifying causal genes (and not causal variants) that impact bulk expression levels, while the latter focuses on identifying causal variants without revealing the underlying mechanisms. Despite these advances, pinpointing causal disease variants, particularly when multiple candidate SNPs are in high linkage disequilibrium (LD), and how they impact causal genes remain challenges.

In this study, we combined sQTL analysis and deep learning-based AI variant annotation to discover causal variants that directly affect splicing. Ninety percent of human genes encode for alternatively spliced transcripts[13], and mutations at splice sites can directly alter expression of transcripts and protein isoforms. It is estimated that up to 60% of rare disease-causing variants exert their pathogenic effects by disrupting normal splicing patterns[14], while the contribution of common variants that affect splicing to complex traits and diseases is comparable to, or even larger than, that of eQTLs[15–17].

Past work has identified sQTLs at baseline in lymphoblastoid cell lines (LCLs)[18], other cell types[3, 19], and complex tissues[20, 21]. Unlike eQTLs, which typically capture aggregated gene-level expression changes, sQTLs can reveal effects of genetic variation on specific molecular events that regulate individual splice junctions that control protein isoforms. However, most sQTL studies do not integrate sQTL identification with systematic variant annotation that reveals causal variants and underlying splice mechanisms.

A major factor that has hampered our understanding of how SNPs affect splicing is incomplete functional annotation, such as undocumented splice events and inaccurate prediction of splice sites. In understanding the effects of rare variants, this has previously left the majority of variants overlapping predicted splice sites categorized as variants of unknown significance (VUS)[22]. Recent application of deep learning algorithms and genome sequences to predict splicing variants, such as SpliceAI[23] and MMSplice[24], has revolutionized the interpretation of rare variants. These tools utilize convolutional neural networks (CNNs) and the genomic context of the surrounding DNA sequence of each focus variant to predict the potential of altering transcript isoform. Newer tools, including Pangolin[25] and AbSplice[26], incorporate both DNA- and transcriptome- based models to predict variant splicing score across different tissues. In 2023, AI-based splicing prediction was recommended as a key criterion for variant pathogenic evidence by the ClinGen SVI Splicing Subgroup[27] for diagnosis of rare and severe genetic disease[28]. Thus, the use of AI in interpretation of rare variants has proven highly successful.

We reasoned that sQTL analysis and AI variant annotation are complementary methods that could pinpoint causal disease variants. While sQTLs capture the regulatory effects of genetic variants across large LD-linked regions, deep-learning-based AI models predict splice-altering potential at single-nucleotide variant resolution. By comparing predicted splicing effects within sQTL association signals, the variants with the strongest predicted impact on splice site usage can be prioritized as putative causal variants.

To test this concept, we adapted our previously developed single-cell GWAS platform (scHi-HOST), which combines single cell RNA-seq (scRNA-seq) and GWAS to rapidly identify eQTLs and SNPs that confer resistance and susceptibility to any stimulus[29]. In this study, we used influenza A virus (IAV), a seasonal epidemic virus and significant burden to global public heath that infects approximately 30 million people every year, with 290,000–650,000 deaths[30]. A previous study demonstrated that IAV infection outcome is heritable[31], and a recent large-scale GWAS (> 270,000 individuals) reported two loci associated with influenza infection[32]. Further, previous work has demonstrated the importance of splicing changes during IAV infection[33–36]. These findings highlighted the important but underexplored role of host genetics and splicing in influenza pathogenesis.

In extending scHi-HOST to identify causal sQTLs, we identified > 43,000 sQTLs during IAV infection and used AI-based splice annotation to connect nearly 40% of sJunctions (junctions associated with an sQTL) to a causal variant with a predicted mechanism of action. A predicted causal variant within the poly (ADP-ribose) polymerase 2 (*PARP2*) gene creates a new splice donor site, resulting in a longer protein isoform with 13 additional amino acids. Amplification of cDNA, sequencing, and western blots of CRISPR-edited cells revealed different isoform expression of PARP2 as predicted. Additionally, scHi-HOST data showed that rs2297616 is associated with IAV burden, suggesting that PARP2 isoform usage regulates IAV infection—a finding confirmed with CRISPR-edited cells. This same variant is associated with herpes simplex virus 1 (HSV-1) infection in UK Biobank data[37], pointing to a more general role in viral infection. More broadly, the causal sQTLs identified by our study are highly enriched in human disease GWAS, highlighting the value of integrating sQTLs and AI for identification of causal variants in human disease.

## Results

### scHi-HOST reveals global changes in splicing induced by IAV and reproducible sQTLs in uninfected and IAV-infected states

**Figure 1:**
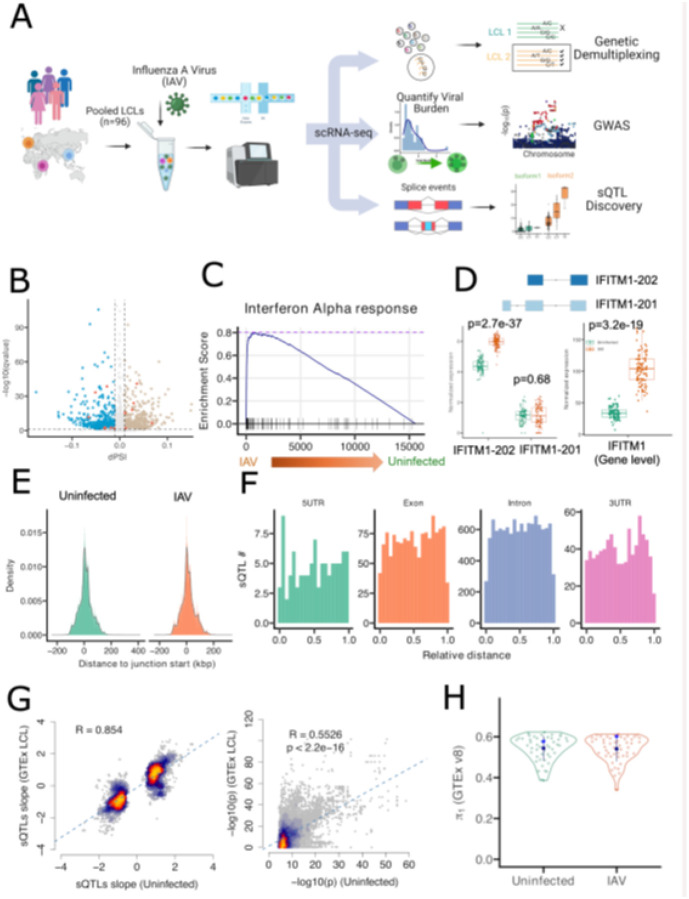
Characterization of global transcript changes after IAV infection and discovery of sQTLs based on scHi-HOST splicing platform. **A)** Flowchart of scHi-HOST sQTL pipeline. **B)**Volcano plot of differentially spliced junctions between uninfected and IAV-infected. Each dot represents a splice junction. The bisque-colored dots indicate significantly higher usage of junctions after IAV (dPSI > 0.01), and blue dots indicate significantly lower usage of junctions after IAV (dPSI < −0.01), gray dots depict junctions that are not significantly different. Orange dots represent splice junctions from genes in the interferon alpha response pathway. **C)** GSEA plot shows significant enrichment of interferon alpha response pathway genes after IAV infection. **D)** The major transcripts of *IFITM1* from the interferon alpha response pathway and boxplots of *IFITM1* expression at the transcript level (left) and gene level (right). **E)** The distribution of sQTLs relative to the location of splicing junctions. The majority of identified sQTLs are located near the junction starting site and decrease quickly with distance. **F)** Distribution of sQTLs across four major gene features. 5’ UTRs, exons, introns, and 3’ UTRs for MANE (Matched Annotation from NCBI and EMBL-EBI) transcripts were scaled from 0 to 1 (to indicate the beginning and end of each feature) and divided into 20 bins. Each sQTL was placed in a bin based on its relative location. **G)** Replication of scHi-HOST sQTLs in GTEx. The left panel shows slope comparison of significant sQTLs from the uninfected scHi-HOST condition (y-axis) and GTEx EBV-infected LCL (x-axis), and p-value comparison (right panel). **H)** Boxplot of statistics indicates scHi-HOST are highly reproducible in GTEx 49 tissues for both Uninfected and IAV.

To characterize the global transcriptional response to IAV infection, we conducted alternative splicing analysis and sQTL discovery in an IAV-infected LCL model based on our single-cell GWAS platform, scHi-HOST[29]. The scHi-HOST workflow consisted of five key components: 1) infection of 96 pooled LCLs from six 1000 Genomes populations with the CA09 IAV strain, followed by scRNA-sequencing, 2) assignment of each singlet to its corresponding LCL by genetic demultiplexing[38], 3) mapping and quantification of reads for generation of pseudobulk phenotypes, 4) association analysis on viral burden phenotypes, and 5) sQTL discovery with Leafcutter[16] (Fig. 1A).

To quantify splicing changes during IAV infection, we counted intron clusters using Leafcutter and identified 33,315 junctions in the uninfected condition and 28,366 junctions under IAV infection. Further differential splicing analysis identified 2,151 junctions from 1,563 genes that were significantly altered between IAV and uninfected conditions (q-value < 0.05 and |ΔPSI| > 0.01) (Fig. 1B; **Table S1)**. Of significant differentially spliced junctions, ~ 62% resulted from exon-skipping events (**Fig. S1**). Gene set enrichment (using Enrichr [39–41]) for genes that harbor significant differentially spliced junctions revealed strong enrichment of interferon-related pathways after IAV infection, including interferon alpha response (Fig. 1C; **Fig. S2; Table S2**). Elevated interferon alpha and gamma responses were visualized at the single-cell level using UMAP plots (**Fig. S3**). Consistent with previous findings[42], we found significantly elevated aggregated *IFITM1* expression of all transcript isoforms after IAV infection (p = 3.2e-19) (Fig. 1D). At the transcript isoform level, the canonical isoform *IFITM1–202*, was dramatically upregulated after infection relative to uninfected controls (p = 2.7e-37). However, the alternative primary isoform, *IFITM1–201*, showed no significant change (p = 0.68) (Fig. 1D). Thus, IAV infection induces higher expression of *IFITM1* by selectively upregulating the *IFITM1–202* isoform rather than the *IFITM1–201* isoform. These findings underscore how transcript analysis enables the capture of isoform-specific changes during pathogen infection.

We used Leafcutter to identify sQTLs that regulate splicing in uninfected and IAV-infected conditions. From uninfected LCLs, we identified 53,292 sQTL-junction pairs from 597 junctions and 317 genes, and from IAV-infected LCLs, we found 43,015 sQTL-junctions affecting 411 junctions from 217 genes (FDR < 0.05; **Fig. S4; Table S3**). Unsurprisingly, increased read depth enhanced detection: genes with high expression showed increased probability of detecting sQTLs, peaking at around 10,000 reads (**Fig. S5**). Therefore, we incorporated library size as a covariate in our sQTL pipeline. Note that mean read depth per gene was higher for uninfected (366) compared to IAV-infected conditions (283), and a greater number of singlets were analyzed in the uninfected state (31,155) compared to IAV-infected (13,794). Both factors likely resulted in greater power to detect sQTLs in uninfected LCLs in our dataset.

We examined the genomic distribution of sQTLs relative to their target splice junctions (sJunctions). As expected, density distribution showed that most sQTLs from both conditions were located close to the junction site and decreased with relative distance (Fig. 1E). 51.2% of uninfected and 51.8% of IAV sQTLs were within 10 kb of alternative splice event, consistent with previous studies that reported that two-thirds of sQTLs fall within a 10 kb window[34]. We next mapped all sQTLs to gene features and visualized their positions after normalizing across the relative length of each gene feature. sQTLs were detected most in introns, followed by intergenic regions, exons, 3’ UTRs, and 5’ UTRs (Fig. 1F; **Fig. S6**). Within each feature, sQTLs were found at similar density throughout the feature type.

To assess the reproducibility of our identified sQTLs, we compared them to sQTLs identified by GTEx[3]. We found both effect size (β) and p-values of matched sQTL-junction pairs were consistent between our uninfected condition and GTEx EBV-transformed LCLs (R2 = 0.854, p < 2.16e-16 for β comparison; R2 = 0.55, p < 2.16e-16 for p-values) (Fig. 1G). We further evaluated the replication of uninfected and IAV sQTLs using the π_1_statistic against all 49 GTEx tissues. We found intermediate-high π_1_ values for both conditions (Fig. 1H) (median value 0.56 for Uninfected, 0.56 for IAV), indicating most of our identified sQTLs were also detected in GTEx.

### sQTLs identified during IAV infection are enriched for genes involved in interferon signaling and can be constitutive, responsive, or induced

**Figure 2:**
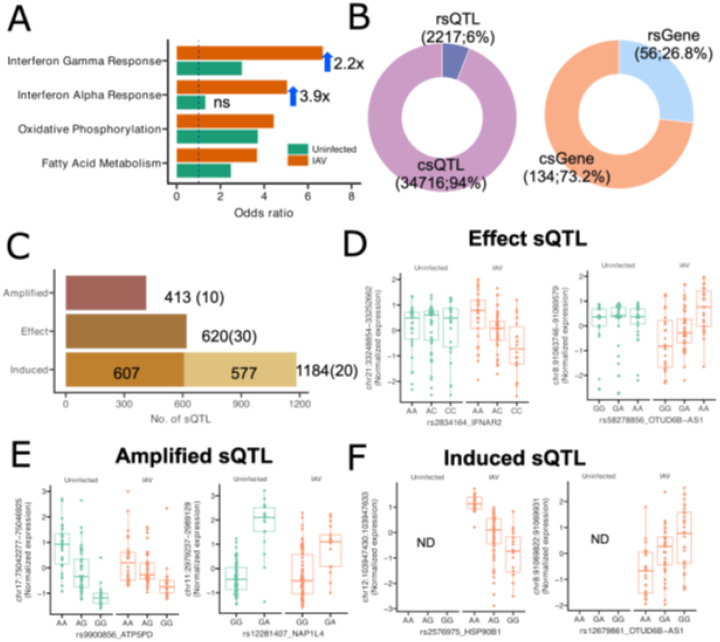
Classification of scHi-HOST IAV sQTLs. **A)** Enrichment analysis of MSigDB Hallmark gene sets based on detected sGenes. Four gene sets at FDR < 0.1 were identified during IAV infection, including interferon gamma and alpha response pathways. **B**) Donut plots illustrating the distribution of IAV sQTLs (left) and sGenes (right). The IAV sQTLs are categorized into two broad groups: IAV-responsive sQTLs (rsQTLs) and constitutive sQTLs (csQTLs). **C**) Barplot depicting three types of IAV rsQTLs. The z-test was employed to define amplified and effect rsQTLs. Induced rsQTLs are exclusively detected in the IAV-infected condition and not detected (n=607) or filtered by Leafcutter due to minimum intron cluster ratio < 0.001 (n=577) in uninfected. **D**) Two examples of effect rsQTLs. SNP genotypes show no correlation with normalized junction expressions under uninfected conditions (β = 0.007, p = 0.94 for *IFNAR2*; β = 0.06, p = 0.63 *OTUD6B-AS1*) but exhibit significant correlation under IAV-infected conditions (β = −0.69, p = 6.3e-7 for *IFNAR2*; β = 0.7, p = 1.7e-7 *OTUD6B-AS1*). **E**) Two examples of amplified rsQTLs. The rsQTLs are significantly correlated with normalized junction expressions in both conditions, but the slope is significantly enhanced or reduced in one condition based on z-test. For *ATP5PD*, β = −1.16, p = 7.8e-14 uninfected; β = −0.66, p = 2.3e-7 IAV-infected. For *NAP1L4*, β = 2.2, p = 6.9e-19 uninfected; β = 1.43, p = 4.95e-13 IAV-infected. **F**) Example of two induced rsQTLs. The junctions were detected in the IAV-infected condition and not detected (ND) under uninfected. For *HSP90B1*, β = −0.99, p = 8.9e-11 IAV-infected. For *OTUD6B-AS1*, β = 0.73, p = 1.5e-7 IAV-infected.

We performed gene enrichment analysis on sGenes to characterize the biological process underlying identified sQTLs based on the MSigDB Hallmark gene set. Four gene sets showed significant enrichment at an FDR level of 0.1, with three of these also showing significant enrichment in uninfected LCLs (Fig. 2A; **Table S4**). Three gene sets, interferon gamma response, oxidative phosphorylation, and fatty acid metabolism, were significant under both conditions, although interferon gamma response showed a 2.2x greater enrichment with IAV infection. Interferon alpha response showed even greater enrichment during IAV infection and was not significantly enriched in the uninfected state. The sGenes found within this gene set include MX1, HLA-C, and IFI44, which play important roles in host response specifically to viral infections. These findings highlight genetic control of splicing in key innate immunity genes that becomes apparent only in the context of infection.

sQTLs identified during IAV infection could be constitutive, responsive, or induced. To differentiate sQTLs that are constitutive compared to those whose effect size changes with infection, we performed a z-test to compare the slopes of matched sQTL-junctions, followed by the FDR correction. Among the 41,831 matched sQTL-junction pairs, 13.3% (n = 2,217) showed a significant difference in slopes (or were not defined in the uninfected condition) and were therefore defined as IAV response sQTLs (rsQTLs), while the remaining 86.7% (n = 34,716) were defined as constitutive sQTLs (csQTLs) (Fig. 2B). The 2,217 rsQTLs affect splicing of 56 rsGenes (26.8%), while the 34,716 csQTLs affect 134 csGenes (73.2%) (Fig. 2B), demonstrating a substantial number of sQTLs exhibit IAV-responsive regulation.

These IAV rsQTLs could be further categorized as amplified, effect, and induced rsQTLs (Fig. 2C). Effect rsQTLs (n = 620; Fig. 2D) are not associated with a junction in one condition but are significantly associated in the other condition, while amplified sQTLs (n = 413; Fig. 2E) are significant sQTLs in both IAV and uninfected conditions but show increased slope in one condition. Finally, some rsQTLs are associated with a junction that is only detected during IAV infection. Such induced rsQTLs (n = 1184; Fig. 2F) are associated with splicing events that appear to only occur during infection. Of these, 607 induced rsQTLs have no matched junctions in uninfected, while the other 577 have extremely low expression of matched junctions and were therefore filtered out by Leafcutter’s default filtering parameter (“--mincluratio 0.001” which indicates the minimum fraction of reads in a cluster that support a junction).

#### Integration of AI with sQTLs reveals putative causal SNPs

We identified a total of 61,216 sQTL-junction pairs, which were associated with only 721 unique splice junctions from 378 sGenes. While typically many sQTLs in high LD are associated with each splicing event, we hypothesized that each independent signal would be driven by a single causal sQTL.

To identify causal variants associated with each splice event, we applied two state-of-the-art AI-based splicing prediction tools, SpliceAI and Pangolin. Both AI models employ convolutional neural networks to estimate splicing changes by comparing predicted splice potential between reference and alternative alleles based on local genomic sequence context. SpliceAI predicts the probability that a nucleotide substitution alters canonical donor or acceptor sites, providing four scores: acceptor gain/loss and donor gain/loss. Pangolin uses a similar deep-learning architecture but predicts splice-site usage with tissue specific context, outputting two probabilities (gain or loss) without distinguishing donor from acceptor sites. Both tools have demonstrated superior accuracy compared to other methods[43], with SpliceAI providing clear donor/acceptor gain/loss interpretability and Pangolin offering tissue-aware prediction and multi-species training data. Therefore, we integrated both methods to obtain a robust set of casual splice variants with a clear mechanistic prediction.

**Figure 3:**
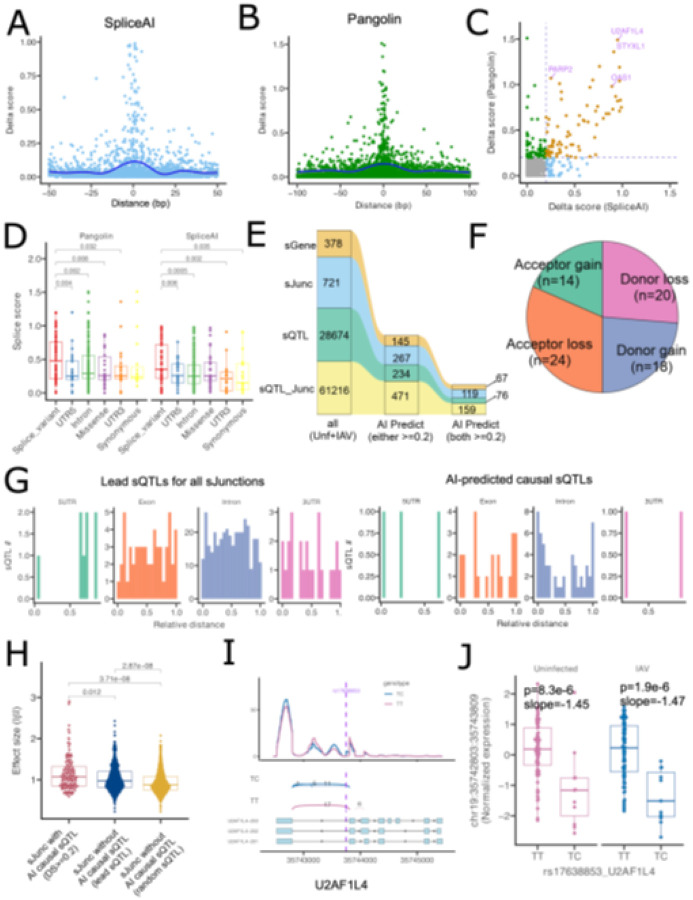
Integration of sQTL identification with AI to identify putative causal variants. **A)** The distribution of SpliceAI delta_scores for scHi-HOST sQTLs plotted based on their location relative to the splice junction. **B)** The distribution of Pangolin delta_scores for scHi-HOST sQTLs plotted based on their location relative to the splice junction. **C)** Scatter plot of SpliceAI delta_score against Pangolin delta_score. The vertical and horizontal line indicate a cutoff of 0.2. The dot color indicates those that are shared (orange), SpliceAI-specific (blue), or Pangolin-specific sQTLs (green). **D)** Boxplot of sQTL splice scores across different groups. The p-values are from the Wilcoxon rank sum test. **E)** River plot summarizes the number of sQTL, sGenes, sJunctions. With a cutoff of 0.2 for both SpliceAI and Pangolin, we identified 76 causal sQTLs. **F)** The mechanism of the 76 causal variants assigned by SpliceAI. **G)** Distribution of lead sQTLs (left) and AI-predicted causal sQTLs (right) across 4 major gene features. 5’UTRs, exons, introns, and 3’ UTRs for MANE (Matched Annotation from NCBI and EMBL-EBI) transcripts were scaled from 0 to 1 (to indicate the beginning and end of each feature) and divided into 20 bins. Each sQTL was placed in a bin based on its relative location. **H)** Boxplot of absolute values of sQTLs from sJunctions with vs without AI causal variants. **I)** Sashimi plot of *U2AF1L4* from pseudo-bulk data stratified by rs17638853 genotype. Exon reads are represented by the peaks in the top and junction reads are represented by arcs in the middle. **J)**Genotype boxplot of rs17638853 against normalized *U2AF1L4* junction counts. Boxplots show median and interquartile range, with each dot representing normalized junction read of a single LCL sample.

As expected, higher delta scores (DS) from both methods were assigned to sQTLs closer to the splice junction, especially within the splice site (distance = 0) or +/− 10 bp for both SpliceAI (Fig. 3A) and Pangolin (Fig. 3B). A direct comparison of the two methods showed high concordance along with some algorithm-specific variants (Fig. 3C). Categorization by variant annotation from Ensembl VEP (Variant Effect Predictor) v106 demonstrated that sQTLs overlapping with known splice sites had the highest predicted splice scores from both tools, significantly higher than other annotated groups, such as 3’ UTR and 5’ UTR (Fig. 3D).

By adopting a DS cutoff of 0.2 from previous studies[23, 25] for both SpliceAI and Pangolin, we identified 76 high-confidence causal variants from 28,674 sQTLs. These causal variants are associated with 67 unique sGenes and 119 sJunctions, indicating that these AI tools agree on the likely causal variant for 18% of sGenes and 17% of sJunctions (Fig. 3E; **Table S3**). If we took a less stringent approach by requiring that only one of the tools have a DS > 0.2, then 38% of sGenes and 37% of sJunctions had a predicted causal variant. Further mechanistic classification of the 76 high-stringency causal variants revealed a slightly higher number of acceptor loss (n = 24, 31.6%) and donor loss (n = 20, 26.3%) events, followed by donor gain (n = 18, 23.9%) and acceptor gain (n = 14, 18.4%) (Fig. 3F). Further, in contrast to sJunction lead variants that had a distribution similar to all sQTLs, AI-identified causal variants follow more of a U-shaped distribution in exons and introns, consistent with causal variants being located near known splice junctions (Fig. 3G; **Fig. S7**; compare to Fig. 1F). Notably, the distribution still displayed many putative causal variants far from known junctions, which may reveal previously unknown junctions or splicing enhancers.

Thus, AI was able to assign a likely causal variant to nearly 40% of sQTL signals. However, this was still substantially less than the 70–90% sensitivity reported in using these same tools to classify rare, splice-disrupting disease variants[27, 43]. We hypothesized that unlike the rare splice-disrupting variants associated with severe disease, common sQTLs likely have less severe effects on splicing and thus would be more challenging for deep learning models to identify. We grouped all sJunctions as having a likely causal variant assigned by AI (SpliceAI and Pangolin scores ≥ 0.2) or not. Comparing the effect sizes (absolute value of β) of AI-predicted causal variants vs. randomly selected sQTLs for each sJunction demonstrated that cases where a causal variant was predicted by AI displayed a larger effect size (Fig. 3H). As the AI-prediction is made independently of effect size, the comparison to randomly select sQTLs was considered appropriate, but causal variant effect sizes were still significantly greater even when compared to lead variants for sJunctions with no strong AI-predicted causal variant. This indicated that these deep learning methods are more likely to identify causal variants for sQTL association loci that have more severe splicing effects (larger effect sizes).

Among the top-ranked variants, rs17638853 had a DS of 0.95 by SpliceAI and of 1.49 by Pangolin. This variant is located within the last intron of *U2AF1L4* (U2 small nuclear RNA auxiliary factor 1-like 4, a paralog of *U2AF1*), which encodes a splicing factor that is part of the heterodimeric complex that recruits the U2 snRNP to the polypyrimidine tract and AG dinucleotide at the 3′ splice site. The alternative allele C creates a novel 5’ donor site based on Sashimi plot and SpliceAI prediction (Fig. 3I), creating a previously unknown isoform that is 19 amino acids longer. This was further confirmed by the AlphaGenome[44] prediction that the alternative allele C is exclusively associated with a shorter junction in three different tissues (Lung, EBV-transformed LCL, and Whole blood (**Fig. S8**). rs17638853 is a constitutive sQTL, as the heterozygous genotype TC was significantly correlated with lower normalized junction ratio in both uninfected and IAV states (Fig. 3J).

Similarly, a causal variant for an sQTL signal for *OAS1* was also identified by both methods (rs10774671; DS of 0.89 by SpliceAI and of 0.98 by Pangolin). The variant determines *OAS1* exon usage (**Fig. S9**). It has been previously reported that the A allele results in expression of only the p42 isoform of OAS1, while the G allele results in expression of both the p46 and p42 isoforms of OAS1[45]. The p46 isoform is prenylated which has been shown to be necessary for OAS1 dsRNA sensing of SARS-CoV-2[46]. This causal variant has undergone further characterization as it is associated with severe COVID-19 and West Nile Virus[45–52]. Collectively, these findings demonstrate the utility of AI-driven splicing predictions in refining sQTL signals to pinpoint causal variants, particular those of larger effect size.

### Experimental validation of a predicted causal variant: rs2297616 creates a new 5’ splice donor site resulting in a longer protein isoform of PARP2

**Figure 4:**
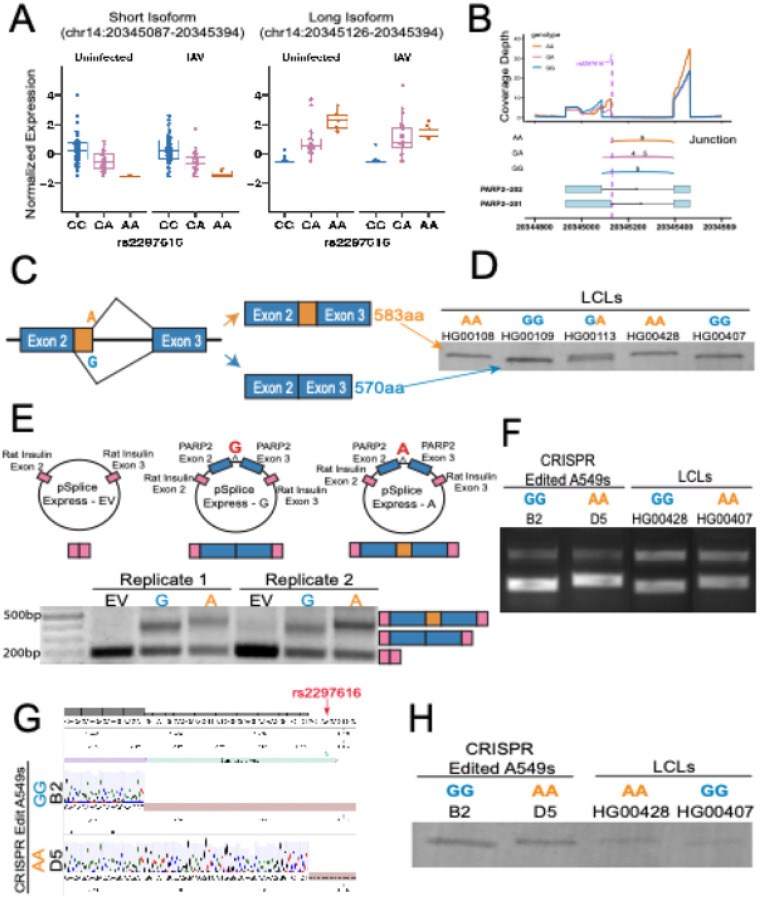
rs2297616 control of 5’ splice donor usage of *PARP2* results in two different protein isoforms. (**A**) Genotypic boxplots of normalized *PARP2* junction reads for long and short isoforms with or without IAV infection. Boxplots show median and interquartile range, with each dot representing normalized junction reads of a single LCL donor. (**B**) Sashimi plot of *PARP2* from pseudo-bulk data stratified by rs2297616 genotype. Exon reads are represented by the peaks in the top plot of coverage depth while junction reads are represented by arcs in the middle plot. These reads can be compared to the two *PARP2* isoforms, represented below these plots. (**C**) Model of predicted splice donor site and protein size based on rs2297616 sQTL data, Sashimi plot, and Maxent scores. (**D**) Protein lysates from LCLs of either AA (HG00108, HG00507), GG (HG00109, HG00428) or GA (HG00113) genotype reveal PARP2 protein size differences that correlate with genotype. PARP2 protein from AA LCLs migrates slower than PARP2 from GG LCLs, consistent with the A allele causing production of a longer isoform. GA individuals demonstrate two different sized PARP2 proteins. (**E**) Agarose gel of cDNA from minigene transfection assay with pSpliceExpress plasmids. pSpliceExpress has nothing inserted between the control rat insulin sequences. pSplice Express-G and pSpliceExpress-A have the DNA sequence from the end of exon 2 to the beginning of exon 3 of *PARP2* inserted with either the rs2297616 G or A allele. Transfection with pSpliceExpress empty vector shows a 200bp band consistent with the rat insulin exon sequence length. Transfection of pSpliceExpress-G reveals a cDNA product at 425bp consistent with a shorter splice product. Transfection with pSpliceExpress-A reveals a product at 464bp which is consistent with a splice donor site and therefore larger splice product. (**F**) cDNA from A549 cells that were edited at rs2297616 to have either the G or A allele show splicing consistent with the minigene assay. D5 cell line (homozygous A) have a longer splice product than B2 cells (homozygous G). The cDNA products align with cDNA from LCLs of either genotype. (**G**) Sequencing of gel extracted cDNA from (F) shows inclusion of the predicted 39 nucleotides in the AA base edited cells. (**H**) Protein lysates from base edited A549 cells reveal expected protein isoforms from genotype. D5 (homozygous A) with a larger PARP2 isoform than B2 cells (homozygous G). PARP2 protein isoform size consistent with that of LCL control protein lysates AA (HG00407) and GG (HG00428).

We experimentally validated the mechanism of one of the strongest scoring causal sQTLs, rs2297616, located at the beginning of intron 2 (+ 4bp) of the *PARP2* gene. PARP2, Poly (ADP-ribose) polymerase 2, is a member of the ART enzyme superfamily, which catalyzes post-translational addition of ADP-ribose to proteins in a NAD + dependent manner. There are 17 PARPs in humans, due to paralogous gene duplication, and several have been shown to play a role during viral infections[53]. rs2297616 is associated with two splice junction events in opposite directions. The G allele is associated with greater levels of the chr14:20345987 − 20345394 event and decreased levels of chr14:20345126–20345394 (Fig. 4A; **Fig. S10**). Examination of splice junctions in a Sashimi plot revealed that the SNP was associated with alternative splicing between exons 2 and 3, appearing to change 5’ splice donor usage (Fig. 4B; as well as in **Fig. S10** from AlphaGenome prediction).

The sQTL data support a model where rs2297616 controls which 5’ splice site donor from exon 2 is used (Fig. 4C): If there is a G at rs2297616, an earlier splice site is used, resulting in a predicted protein 570 amino acids in length. However, if there is an A at rs2297616, an alternative splice donor site is created, resulting in a protein 583 amino acids in length. These protein isoforms are the two known common isoforms (Q9UGN5–1 (583 amino acids) and Q9UGN5–2 (570 amino acids), but how their expression is regulated was previously unknown. Maximum entropy modeling supported this predicted splice site change, with the A allele having a greater Maxent score (9.45) than the G allele (6.77), indicative of increased potential for U1 snRNP binding[54].

Western blot of LCLs confirmed that PARP2 isoform size was genotype dependent. LCLs from GG individuals demonstrate a single smaller band than AA, while heterozygous (GA) LCLs reveal two bands of both isoform sizes (Fig. 4D). To determine if the difference in protein size was directly controlled by rs2297616 genotype, we constructed mini-gene splicing reporter plasmids for *PARP2* exons 2–3 with the intervening intron harboring the two distinct alleles (Fig. 4E). PCR of cDNA collected from cells transfected with either splicing reporter revealed different size splice products. The pSpliceExpress plasmid with the A allele revealed a larger band than the pSpliceExpress-G plasmid (Fig. 4E). Further, Sanger sequencing of these bands revealed an additional 39 nucleotides with the A allele—exactly matching the predicted alternative 5’ splice site donor usage (**Fig. S11**).

To further investigate how rs2297616 controls splicing and isoform usage of PARP2, we generated CRISPR-Cas9-edited cell lines using homology-directed repair from lung epithelial cells (A549s, which are naturally homozygous G at this locus). Amplification of cDNA from edited cell line clones D5 (homozygous A) and B2 (homozygous G) revealed a difference in cDNA size, similar to that seen with the pSpliceExpress-A and pSpliceExpress-G plasmids (Fig. 4F). Sequencing confirmed the inclusion of the expected 39 nucleotides in the D5 edited cell line, indicating a later splice site was used (Fig. 4G). Finally, these changes in cDNA size resulted in the expected PARP2 protein size difference by western blot (Fig. 4H). These results show that rs2297616 controls the 5’ splice donor usage of exon 2 in *PARP2* to dictate which protein isoform is expressed.

#### PARP2 rs2297616 is associated with IAV burden

**Figure 5:**
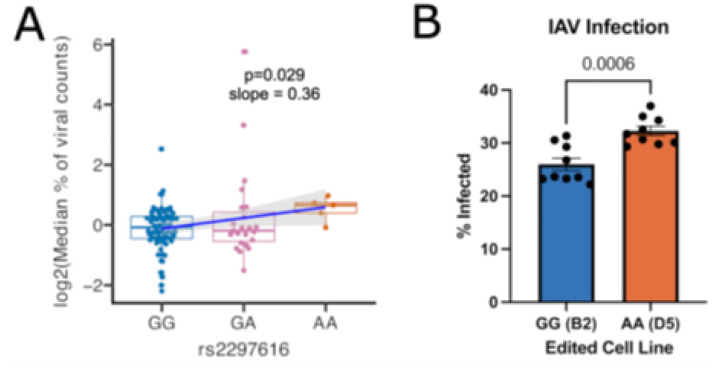
PARP2 rs2297616 A-associated long isoform is more susceptible to CA09 IAV infection. **A)**Genotypic boxplot of median percentage of viral counts for rs2297616. The AA genotype is significantly associated with higher median percentage of viral counts. **B)** IAV infection of CRISPR-Cas9-edited A549s read out by flow cytometry confirms A-associated long isoform is more susceptible to IAV infection.

Previous studies have implicated PARPs as regulators of IAV infection[55–58]. To investigate whether rs2297616 affects influenza infection, we examined its association with CA09 IAV burden, as measured by median viral count of all single cells for each LCL in our scHi-HOST data. The individuals carrying the rs2297616 AA genotype exhibited modestly higher median viral burden compared to those with GA or GG genotypes (Fig. 5A; p = 0.029), suggesting that the A allele (the derived allele causing the longer isoform) likely enhances susceptibility to viral replication.

We tested this hypothesis with our edited A549s. IAV infection of these cell lines showed an effect on viral burden consistent with the sQTL association. The homozygous A edited cell line (D5) showed a significantly higher proportion of infected cells compared to homozygous G cells (B2), further confirming the A allele increases susceptibility to IAV infection (Fig. 5B; p = 0.0006).

### scHi-HOST causal sQTLs are associated with diverse human diseases.

**Figure 6:**
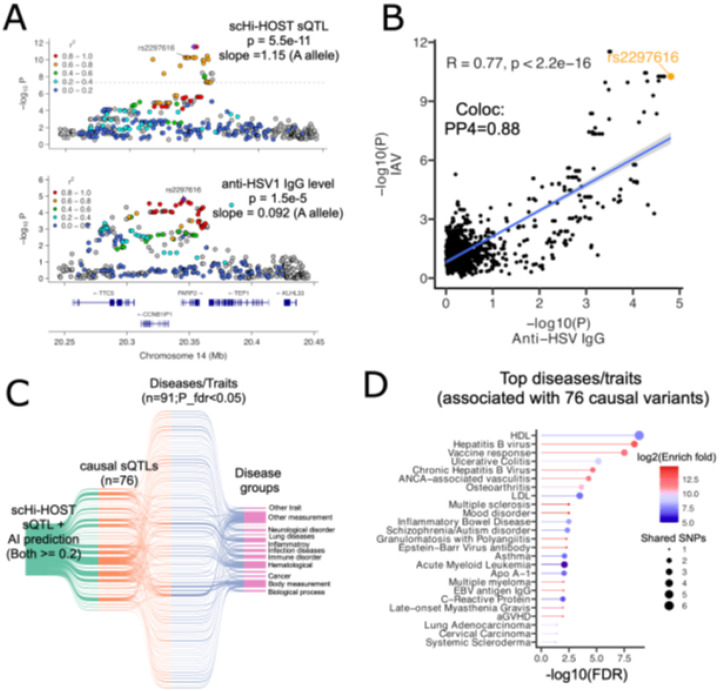
scHi-HOST sQTL is associated with various human diseases. **A)** LocusZoom plots of PARP2 sQTL in IAV (this study) and GWAS of anti-HSV1 IgG level. **B)** Coloc analysis and p-value comparison of PARP2 sQTL and GWAS of anti-HSV1 IgG level. **C)** Sankey plot of 76 causal sQTLs based on output of iCPAGdb analysis. **D)** Dotplot shows top human diseases that are associated with 76 causal sQTLs. The dot color depicts the significance of p-values, and dot size represents the degree of fold enrichment.

To investigate whether the rs2297616 impacts viral infections beyond IAV, we searched the previous web browser of OpenGWAS[59] and found that it has a moderate association (p = 1.5e-5) with anti-HSV1 (herpes simplex virus 1) IgG levels with concordant directionality, then downloaded the full GWAS summary statistics for the study[37] from the NHGRI/EBI GWAS catalog[60]. The A allele, which is associated with higher IAV burden, is also associated with greater risk of HSV1 based on antibody measurement. To test whether the sQTL effect on *PARP2* and the HSV-1 association likely shared a causal variant, we performed colocalization analysis using coloc[9]. The *PARP2* sQTL signal showed strong overlap with the GWAS signal, and rs2297616 was the lead variant for the HSV-1 IgG signal (Fig. 6A). Colocalization was demonstrated by a PP4 of 0.88, and we found significant positive correlation of −log p-values SNPs at this locus for the two traits (R = 0.77, p-value < 2.2e-16) (Fig. 6B; **Table S5**). This shared causality at rs2297616 suggests that alternative splicing of *PARP2* may contribute to interindividual difference in antiviral responses to multiple viral pathogens.

To assess whether other causal sQTL variants were associated with human disease, we performed cross-phenotype analysis for the 76 high-confidence causal sQTLs using our previously developed tool, iCPAGdb[61]. iCPAGdb estimates disease associations based the shared genetic variants using a LD-aware framework and GWAS loci cataloged by the NHGRI-EBI GWAS catalog[60], encompassing variants from more than 4400 diseases. This comprehensive integrative analysis demonstrated that the 76 causal sQTLs were associated more than expected by chance with 91 different diseases/traits (Fig. 6C; **Table S6**). Cross-trait analysis revealed these variants are often significantly associated with multiple diseases, including infectious, autoimmune, and metabolic disorders (Fig. 6D). Top associations included hepatitis B virus infection, Epstein–Barr virus infection, asthma, and multiple sclerosis.

Together, these results demonstrate that the integration of single-cell sQTL mapping with AI-based variant prioritization provides a powerful framework for uncovering causal splicing variants underlying complex human diseases.

## Discussion

In this study, we developed a systematic and efficient approach that combines single cell-based sQTL detection with AI-based splice prediction to identify causal splice variants. Using our scHi-HOST single-cell GWAS platform with uninfected and IAV-infected LCLs, we identified 28,674 sQTLs from 378 sGenes and localized underlying causal variants to 76 SNPs with deep-learning-based splice prediction. These findings also build upon previous studies of the effects of IAV on host splicing[35, 36] and identification of sQTL in the context of IAV infection[33, 34] that did not examine the impact of sQTLs on IAV infection phenotypes or systematic AI-based identification of causal variants as we have now done.

Deep-learning models, such as SpliceAI and Pangolin, leverage sequence information (with Pangolin additionally trained on multi-species data) to infer the effects of variants on canonical and cryptic splice sites, whereas sQTLs quantify how genetic variants are associated with splicing events in populations. The convergence of these complementary signals strengthens causal inference and enables the discovery of common variants that mechanistically alter splicing and contribute to complex disease risk. Only 19.7% of the 76 causal variants and 13.2% of the 234 less stringent causal variants were also the lead variant for the sQTL signal. Further, as molQTL and GWAS lead variants are often in strong LD with many other SNPs, the integration of QTL and AI demonstrated here is complementary to colocalization approaches in identifying causal variants that underlie molQTL and GWAS signals and revealing precise mechanisms of action.

The current deep learning-based AI models for splicing have mainly been applied to predicting the impact of rare, splice-disrupting variants[27, 28]. AI has been quite successful in this application, achieving up to 90% sensitivity[43] and being incorporated into clinical genetic workflows[27]. Using deep learning to systematically discover *common* genetic causal variants influencing alternative splicing has received less attention. We found that nearly 40% of sJunctions can be assigned an sQTL causal variant by at least one of the splicing AI tools. On average, these causal variants are associated with splicing differences of larger effect size, consistent with the idea these deep learning tools are better at identifying more severe changes that are perhaps under less evolutionary constraint. However, this hypothesis needs further systematic evaluation in future work.

Each of the 76 high confidence causal variants has a concordant AI-generated hypothesis from both Splice-AI and Pangolin that suggests how 5’ or 3’ donor/acceptor sites are impacted by the variant. These 76 include an *OAS1* sQTL variant that was identified 20 years ago[45] but has undergone increased interest due to its association with severe COVID-19 [46–48, 50–52].

We experimentally validated the mechanistic effect of rs2297616 on *PARP2*, which ranked 5th in splice score prediction. Our analysis and experiments defined how the derived A allele creates a 5’ donor site at the end of exon 2 which lengthens the transcript by 39 nucleotides, therefore creating a PARP2 protein isoform 13 amino acids greater in length. While earlier studies linked rs2297616 genotype to alternative *PARP2* splicing[62, 63] using splicing-sensitive microarray data in HapMap CEU population, we have provided the first evidence that connects rs2297616 genotype to differing PARP2 protein isoform expression and used CRISPR editing to stringently assess the effect of this variant in its normal genomic context. Furthermore, we have gone on to connect PARP2 protein isoform expression to IAV susceptibility. Together, these experiments elucidate mechanism from genotype to splice site alteration to protein isoform to viral replication. Our data demonstrates that combining sQTL, AI splicing prediction, and cellular genetic association in scHi-HOST can provide a direct mechanism by which a single nucleotide modulates susceptibility to infection.

PARPs have long been implicated in multiple viral infections, with both reported pro-viral and anti-viral roles. PARP11 and PARP12 have been shown to play a synergistic anti-viral role against Zika virus[64], while PARP12 and PARP14 increase IFN production during mouse hepatitis virus infection[65]. Poly(ADP-ribosyl)ation is induced upon HSV-1 infection and blocked by PARP1/2 inhibition[66]. During IAV infection, PARP1 has been shown by multiple studies to enhance IAV replication[56, 57, 67]. In contrast, PARP13 has been revealed as an antiviral factor against IAV[68] and a recent preprint has shown that all PARPs exhibit antiviral activity against IAV when overexpressed[58]. Since overexpression studies usually examine a single protein isoform, our findings highlight the importance of evaluating isoform usage in infection outcomes, especially in regard to PARP proteins as previous studies have suggested[69]. For PARP2, this extends beyond IAV infection as we have found that the same sQTL locus colocalized with anti-HSV-1 IgG, indicating that these two PARP2 isoforms have broader roles during viral infection that will be investigated mechanistically in future work.

Our study has limitations. First, the dataset used in this study includes only 96 samples from six global populations, which may limit power for discovering sQTLs of more modest effect. Expanding to more diverse ancestry samples and additional cell types, will enhance sQTL discovery by inclusion of lower frequency variants and cell-type specific splicing junctions. Second, the junction readouts are based on short read sequencing which rely on algorithmic calling. Long-read sequencing would improve sQTL mapping by directly quantifying full-length transcript isoforms and reducing the error of junction readouts in complex sequence regions, such as segmental duplications.

## Conclusions

Our study provides 1) a catalog of causal cis-sQTLs with predicted mechanism of action, 2) experimental validation of an sQTL that changes PARP2 protein length to regulate IAV infection in cells, and 3) a generalizable framework for moving from association to mechanism for complex traits. These insights highlight how combining sQTL mapping, AI, and functional validation can enhance discovery of disease causal variants with a clear genetic mechanism of differential splicing, enabling possible therapeutic and diagnostic strategies.

## Methods

### Cell lines

Lymphoblastoid cell lines (LCLs; EBV-immortalized B cells) from the 1000 Genomes Project were purchased from the Coriell Institute. LCLs from 6 populations were included, LWK (Luhya in Webuye, Kenya), ESN (Esan in Nigeria), GBR (British in England and Scotland), IBS (Iberian in Spain), CHS (Han Chinese South), and KHV (Kinh in Ho Chi Minh City, Vietnam). LCLs were maintained at 37°C in a 5% CO_2_ atmosphere and were grown in RPMI 1640 media (Gibco #21870) supplemented with 10% heatinactivated fetal bovine serum (HI-FBS, Gibco #10082), 2 mM L-glutamine (Gibco #25030081), and 100 U/ml Penicillin-G, and 100 mg/mL Streptomycin (Gibco #15140122).

A549 cells (ATCC) were grown in DMEM + 10% FBS + 1% Pen-Strep.

### Influenza A virus infections

The CA09 (California/7/09, H1N1) sfGFP-HA strain[70] was propagated in MDCK cells and used to infect LCLs and A549 cells. Briefly, virus was used to infect MDCK cells at a low MOI in Opti-MEM-based media containing TPCK-treated trypsin, and nascent virus containing supernatant was collected 48–72hr post-initial infection [71]. To increase the number of infectious units per volume, virions were concentrated prior to use via centrifugation over a sucrose cushion as previously described [72].

For scHi-HOST, LCLs were infected in two groups of 48 LCLs, scHi-HOST Neo (previously scHH-EIK in[29]) and scHi-HOST Morpheus (previously scHH-LGC in [29]). LCLs were pooled in equal numbers and 250,000 total cells were plated in each well of a 24-well plate in PBS with 0.35% BSA, 2 mM glutamine, 100 U/mL penicillin-G, and 100 mg/mL streptomycin. Cells were infected at MOI 25 or left as uninfected controls. At 3 h post infection, each well was spiked with 600 μL of RPMI 1640 media (Invitrogen) supplemented with 10% fetal bovine serum (FBS), 2 mM glutamine, 100 U/mL penicillin-G, and 100 mg/mL streptomycin. At 24 h post infection, cells from each sample were collected, spun down, and resuspended in PBS with 0.04% BSA for single-cell cDNA library preparation.

For A549 infection experiments, A549 cells were plated at 20,000 cells per well in 100 μL growth media (DMEM + 10% FBS + 1% Pen-Strep) in TC-treated 96 well plates. 1 day after plating, media was removed and replaced with 50 μL PBS with 0.35% BSA, 2 mM glutamine, 100 U/mL penicillin-G, and 100 mg/mL streptomycin. Cells were then infected with at an MOI of 10. 1hpi media was removed and replaced with 50uL of Opti-mem + 0.01% FBS + 1% Pen-Strep + 0.35% BSA. 24hpi cells were trypsinized and assayed for percent GFP + using a Guava Easycyte flow cytometer.

### Single cell RNA-seq library preparation, sequencing, and alignment

LCLs were counted using a Guava EasyCyte HT with 7-AAD staining. The scRNA-seq cDNA library was barcoded and prepared using the 10x Chromium Single Cell 3’ Library and Gel Bead Kits v3.1 (Pleasanton, CA). The scRNA-sequencing resulted in 31,155 and 13,794 cells for uninfected (scHi-HOST NeoUninfected 11180 + scHi-HOST MorpheusUninfected 19975) and CA09 IAV (scHi-HOST NeoCA09 8861 + scHi-HOST MorpheusCA09 4933).

cDNAs were sequenced on an Illumina NovaSeq S4 flow well, with a targeting depth of 100,000 reads per barcoded droplet for infected library. The details of sequencing information for uninfected can be found in our previous publication[29].

Paired-end short reads were mapped to a custom reference genome using *CellRanger* v7.0.1[73] with default parameters. The custom reference genome combined human GRCh38 reference genome and influenza A/California/7/09 reference genome. Sequencing was conducted in two batches, with each of 48 LCLs. For scHi-HOST Neo, the library was single-indexed, and index-hopped reads were removed using 10x Genomics Index-hopping-filter software (https://github.com/10XGenomics/index_hopping_filter)) before aligning to the custom reference genome. For scHi-HOST Morpheus, the library was dual-indexed. Reads were sequenced with read 1 length of 28 base pairs (bp) and read 2 length of 150 bp.

Each droplet was assigned to individual LCL identity using Demuxlet[31]. Demuxlet estimates the likelihood of each droplet identity based on natural genetic variations, matching the SNPs from mRNA reads with individual LCL genotypes. The input bam file of Demuxlet was directly taken from *CellRanger* output. The other input file included a VCF file that contained genotypes for 96. We used Subset-Bam software (10X Genomics) to extract reads from each CellRanger alignment bam into 96 pseudobulk bam files. Pseudobulk bam files were further used for sQTL detection, viral burden quantification, and other analysis.

### LCLs genotypes

LCL genotypes were obtained from the high coverage whole genome sequencing (30x) of 1000 genome project phase 3, generated by New York Genome Center (NYGC; download link: https://www.internationalgenome.org/data-portal/data-collection/30x-grch38). The bcftools v1.9[74] was used to prepare the VCF genotype file and then filtered the VCF file with parameters of “-q 0.05:minor -m 2 -M 2”, to restrict our analysis to only biallelic SNPs and those with minor allele frequency more than 0.05. Genotypic principal components were calculated using Plink v1.9 [75].

### Identification of splicing QTLs (sQTLs)

We performed splicing QTL (sQTL) analysis using an annotation-free pipeline, *Leafcutter* v0.2.9[16], adapted from the GTEx v8 pipeline (https://github.com/broadinstitute/gtex-pipeline). Transcript junction annotations were preprocessed by collapsing isoforms per gene using collapse_annotation.py based on from GTF file from GENCODE v32. RegTools (v0.5.1)[76] was employed to count junction reads for each LCL based on pseudo-bulk BAM file from CellRanger output. The following default parameters was used by RegTools: minimum anchor length of 8 bp (“-a 8”), intron size between 50 bp and 500,000 bp (“-m 50 - M 500,000”), and unstranded mode (“-s 0”). Leafcutter “leafcutter_cluster_regtools.py“ was used for intron clustering for extracted splice junctions, with parameters of filtering out clusters with < 30 split reads (“--min_clu_reads 30”), the minimum fraction of reads supporting junctions in a cluster of 0.001 (“--min_clu_ratio 0.001”), and maximum intron length of 500,000 (“--max_intron_len 500,000”). Leafcutter “prepare_phenotype_table.py” was used to convert intron reads to splice junction ratio and perform quantile normalization. To capture latent confounding factors underlying the junction ratio matrix, we calculated probabilistic estimation of expression residuals (PEER) factors using R peer v1.3[77] based on the normalized junction ratio matrix.

To assess the impact of read depth on sQTL detection, we binned genes by expression level and computed the probability of detecting ≥ 1 sQTL (FDR < 0.05). The probability of detecting a sQTL was defined as the fraction of genes in each bin associated with at least 1 sQTL. This probability rose with expression, peaking at ~ 10,000 reads (**Fig. S3**). Mean read depth per gene was 366 (uninfected) and 283 (IAV), defined as 1x. At 1x, sQTL detection probability was ~ 2.5%, dropping to 0.4% at 0.1x and rising to ~ 7% at 10x, with a plateau near 10x. This indicates that higher read depth enhances sQTL detection.

We then used TensorQTL (v1.0.6) [78] for sQTL mapping based on the phenotype bed files generated from Leafcutter. The SNPs located within 100,000 bp window of a gene’s starting and/or end position were included for the SNP-junction association testing. To account for potential cofounding factors, the first two genotypic principal components (PCs), sex, and top 3 PEER factors were incorporated in the regression model. As some PEER factors were correlated (**Fig. S12**), we evaluated pairwise correlations among the top 10 PEER factors using the R “cor” function. With a p-value cutoff of 0.05, we retained the top three PEER factors. The remaining seven PEER factors were correlated with these top three factors in both uninfected and IAV conditions.

For sQTL analysis, nominal p-values were computed for all SNP-junction pairs from TensorQTL[78]. To account for linkage disequilibrium among variants for individual junction, empirical p-value for the most significant SNP per junction was calculated under the β distribution approximation using the adaptive permutations with parameters of “--permute 1000 10000”. The Benjamini-Hochberg false discovery rate (FDR) method was applied to identify significant splicing genes (sGenes) at an FDR level of 0.05. The significance of sQTL per sGene was defined by comparing their nominal p value to the nominal p-value threshold of the matching sGene.

### IAV response sQTL

We applied a z-test approach to define IAV response sQTL (rsQTL) as described in previous work[79, 80]. The z-test compares the slope difference of a sQTL-junction pair between uninfected and IAV conditions, where the slope values were extracted from TensorQTL output. The equation we used was as follows:

$$z=\frac{\text{slop}e_{\text{IAV}}-\text{slop}e_{\text{uninfected}}}{\sqrt{\text{slope}\_se_{\text{IAV}}+}\text{slope}\_se_{\text{uninfected}}}$$

The resulting p-value were further corrected using Benjamini-Hochberg method from R “p.adjust” function. SNPs with a corrected p-value < 0.05 were defined as IAV response sQTL.

We further grouped rsQTLs into three different categories: effect rsQTL, amplified rsQTL, and induced rsQTL. The effect rsQTL is defined if a SNP is significant after Benjamini-Hochberg correction in the IAV condition, but not in uninfected, and the p-value of slope comparison is < 0.05. The amplified rsQTL was defined if the slope difference of the variant-junction was significant between two conditions and the slopes are in the same direction. Induced rsQTLs are defined as SNP that are significant under the IAV condition, but its significance was not tested in the uninfected condition due to low or no expression.

### Differential splicing genes (DSGs)

Differential splicing analysis was tested using the Leafcutter’s *leafcutter_ds.R* function, incorporating sex as a covariate. Leafcutter employs a likelihood ratio test to compare differences in intron usage between IAV-infected LCLs and uninfected controls. Junctions with a false discovery rate (FDR) ≤ 0.05 were considered significant. The significant junctions were subsequently annotated to their associated genes to facilitate functional enrichment and downstream analysis.

### Annotation of alternative splicing event

The R *psichomics* package[81] was employed to annotate the Leafcutter junction. The hg38 genomic coordinates of these junctions were extracted as input. The resulting annotation includes skipped exon (SE), mutually exclusive exons (MXE), alternative first exon (AFE), alternative last exon (ALE), alternative 5’ splice site (A5SS), alternative 3’ splice site (A3SS).

### Enrichment of sQTLs to GTEx

We used π_1_ statistic[82] to estimate sQTL enrichment in GTEx sQTL datasets for all 49 tissues (v8). For each SNP-junction pair from scHi-HOST, the nominal p-value was extracted from the matched pair in GTEx. Then we used the R *qvalue* package to compute π_0_, and the fraction of true replicated sQTLs or enrichment degree π_1_ is defined as 1- π_0_. As some SNP-junction pairs are not tested or missing in GTEx sQTL, a random p value was assigned from uniform distribution.

### Genetic association for flu burden

Short reads were mapped to the influenza A/California/07/2009(H1N1) genome (GenBank accession: GCA_001343785.1) using CellRanger v7. To remove possible ambient RNA contamination of IAV reads, we used CellBender v.0.3.0[83] with “--fpr 0.01” and “--epochs 150”. CellBender uses a deep generative model to learn a background noise profile to delineate cell-containing and empty droplets and provide noise-free gene count quantification. To identify IAV burden phenotypes, the percentage of viral reads to total reads per cell was calculated. The median viral percentage of reads across all cells per LCL was then used as a pseudobulk phenotype. Genetic association testing was performed using EMMAX[84], which uses a linear mixed model and accounts for population stratification and cryptic relatedness by incorporating pairwise kinship across samples. The kinship matrix was calculated based on autosomal SNPs with minor allele frequency (MAF) > 5%. We incorporated biological sex as a covariate to account for possible sex differences.

### Variant functional annotation

We used Ensembl variant effect predictor (VEP v106)[85] to annotate all genomics variant SNPs using parameters “--everything --offline --check-existing.” Broadly, variants are grouped by their relative location into the following types: introns, exons, 3’ UTR, 5’ UTR, and intergenic, or by functions, such as, missense, synonymous mutation, frame shift, splicing donor or acceptor, or splicing region (1–3 bp of exon or 3–8 bp of the intron) etc.

### Transcript quantification

To quantify abundance of individual transcripts per gene, we re-mapped short reads for each LCL to GRCh38 using *Salmon*[86]. The transcript abundance was imported and normalized using R *tximport*[87] and *DESeq2*[88] package. Raw counts were log-transformed with the addition of one pseudo-count.

### AI-based splicing prediction

We applied both SpliceAI[23] and Pangolin[25] to predict the splicing impact of genetic variants and to annotate the sQTLs. For SpliceAI, we downloaded the pre-computed “genome_scores_v1.3” table from the Illumina BaseSpace platform (https://basespace.illumina.com/). This pre-computed tables provide genome-wide delta scores (DS) and delta distance for all possible SNV and indels based on GRCh38 reference, representing the predicted potential that a variant alters acceptor or donor splice sites[23].

For the Pangolin analysis, we installed Pangolin v1.0.2 locally and ran predictions using a ± 100 bp window around each variant (i.e., the “-d 100” option). For each individual variant, Pangolin estimates the maximum predicted gain and maximum predicted loss in splice-site usage within the given window.

For both Pangolin and SpliceAI we used the “delta score”, defined as the difference between reference and variant allele predictions, as the key indicator of splicing. A higher delta score implies a greater likelihood of a splice-impacting event. As suggested by the Pangolin authors[25], we adopted a delta score > 0.2 from either tool as a robust indicator that the variant may alter splicing.

To further evaluate the splicing effects of sQTLs in independent datasets, we utilized the recently released AlphaGenome software (v0.4) [44]. AlphaGenome implements the S2F (Sequence-to-Function) deep learning model, which is optimized for predicting the molecular consequences of genetic variants on transcriptome-level features with integration of large-scale cohorts of RNA-seq datasets. All predictions were performed using the human GRCh38 reference genome and gene annotation of GENCODE v46. Two ontology terms, EFO:0000572 and EFO:0008952, were used in the analysis, matching to GTEx EBV-transformed LCL and Lung. For each variant, AlphaGenome extracted the surrounding 1 Mbp genomic sequence and generated predictions for both the reference and alternate alleles. The model provides estimates across multiple publicly available RNA-seq datasets (e.g. GTEx), including predicted splicing effects, splice-site usage, and splice-junction counts. The splice effect of variant and splice junction were visualized using AlphaGenome “plot_components” module.

### Connecting causal sQTL to human diseases/traits

To explore potential shared causal genetic effects between sQTLs and public GWAS signals, we performed two independent approaches. First, we performed colocalization analysis using the R package *coloc*[9]. The *coloc* package implements a Bayesian framework to assess the probability of colocalization by integrating summary statistics from QTL and GWAS datasets. We applied default prior parameters (p_1_ = 1 × 10^−4^, p_2_ = 1 × 10^−4^, and p_12_ = 1 × 10^−4^) during the analysis. Evidence of colocalization was defined as a posterior probability of colocalization (PP4) greater than 0.8, indicating a high likelihood of shared causal variants between the sQTL and GWAS signals. LocusZoom visualization for *coloc* results were plotted using the *Locuszoomr* R package[89].

As an alternative approach, we performed a systematic cross-phenotype analysis using our previous developed tool, iCPAGdb[61]. This tool connects user’s SNP set to over 4400 traits from NHGRI/EBI GWAS Catalog using a LD-aware approach. European from the 1000 Genome project was chosen for LD reference. The p-values were corrected from multiple test correction using R “p.adjust” method.

### Immunoblotting

Cells were lysed with RIPA lysis buffer: 50mM Tris-HCl pH 7.4, 150mM NaCl, 0.1% SDS, 0.5% NaDeoxycholate, 1% Triton X-100, cOmplete Mini protease inhibitor cocktail (Millipore Sigma #11836170001) 10mM NaF, and 1-mM Na orthovanadate. SDS-PAGE was performed using MiniPROTEAN TGX Stain-Free Precast 4%–20% gels (Bio-Rad #456–8096). The gels’ stain-free dye was activated by a 5-min UV exposure and protein was transferred to Immun-Blot low-fluorescence PVDF membrane (Bio-Rad #162–0264) using Hoefer TE77X. Blots were developed using LI-COR infrared secondary antibodies (IRDye 800CW Donkey anti-rabbit IgG) and imaged on an LI-COR Odyssey Classic. Primary antibodies used: anti-PARP2 pAb (Active Motif RRID: AB_2793328).

### Minigene Cloning and Transfection

The backbone minigene plasmid, pSpliceExpress, was a gift from Stefan Stamm (Addgene plasmid # 32485; http://n2t.net/addgene:32485; RRID:Addgene_32485) [90]. DNA was extracted from LCL HG00428 and PCR was performed with primers attb1F and attb2R in **Table S7** and Phusion DNA polymerase (Neb). A Gateway BP clonase II reaction (Thermo Fisher) was then preformed overnight at 25C. Site-directed mutagenesis was carried out using Quikchange II XL Site-Directed Mutagenesis Kit (Agilent, #200521) (primers in **Table S7**). A549 cells were seeded at 1.05 × 10^5^ cells per well in a 24 well TC treated plate 24hrs before transfection. Transfection was accomplished with Lipofectamine 3000 (Thermo Fisher, Catalog #L3000008). Cells were harvested 48 hours post transfection.

### Cas9-RNP Editing of A549s

To assemble RNP complexes, we utilized a previously described method[91], with the following modifications for A549 cell type and rs2297616. Three gRNAs for rs2297616 were designed based on GGC site proximity (**Table S7**). Centrifugation was performed at 400 × g for 5 minutes. Both homologous DNA repair fragments (IDT, Alt-R Donor Oligo, 2nmol) were added to each RNP tube at a concentration of 2.5uM each from a 20uM stock (**Table S7**). Each reaction was pulsed twice at 1200V for 30 ms. Cells were then transferred into 500 μL of DMEM + 10% FBS media to incubate for 48 hours at 37Cs. Cells were then split into a TC-treated 6-well plate and DNA was extracted with QuickExtract (Bioresearch Technologies, QE0905T) from each pool. PCR for confirmation of SNP edit was performed using DNA polymerase Phusion (New England Biosciences, M0530S) with primers rs2297616_F and rs2297616_R (**Table S7**) at the following cycle conditions: 1X 98°C 30s, 30X 98°C 10s, 70°C 45s, 72°C 30s, 1X 72°C 10min. PCR product was sent for sanger sequencing (Azenta) with primer: rs2297616_F (**Table S7**) and edit frequency for each pool was evaluated by ICE Analysis (EditCo Bio Inc.). Pool from gRNA1 had the highest edit frequency of 15%. Cells from gRNA1 pool were then plated at a concentration of 0.5cells per well in TC-treated 96 well plates for clonal expansion. Two weeks post seeding cells were expanded for DNA collection with QuickExtract (Bioresearch Technologies, QE0905T). PCR product was sent for sanger sequencing (Azenta) with primer: rs2297616_F (**Table S7**) and edit was visualized with Benchling, https://benchling.com.

### RNA extraction, cDNA synthesis and gel electrophoresis

RNA was extracted using Qiagen kit according to manufacturer’s instructions (###). cDNA was made from 500ng of RNA with iScript (##). PCR products were examined by electrophoresis at 100 V for 30 min in a 2% (w/v) agarose gel with 0.5ug/mL ethidium bromide in 1 × TAE buffer. Gel extractions were preformed with Qiagen kit (##) then analyzed by Sanger sequencing with primer rs2297616_F.

## Supplementary Material

Supplementary Files

This is a list of supplementary files associated with this preprint. Click to download.

- FigSupp20251213v6.pdf
- TableS1IAVvsUninfectedDifferentialSpliceJunction.xlsx
- TableS2differentialsplicegeneHallmarkenrichment.xlsx
- TableS3sQTLsUnfCA09.xlsx
- TableS4sGeneenrichmentMsigDBhallmark.xlsx
- TableS5colocPARP2vsHSV1rs2297616PP4.csv
- TableS6iCPAGdb.xlsx

## Data Availability

Most of the data generated or analyzed during this study are included in this published article and its supplementary information files. Data that are too large to include as supplementary files (scRNA-seq files) will be made available from GEO repository upon publication.
